# Microbial and Quality Attributes of Beef Steaks under High-CO_2_ Packaging: Emitter Pads versus Gas Flushing

**DOI:** 10.3390/foods13182913

**Published:** 2024-09-14

**Authors:** Seyed Mohammad Hassan Mortazavi, Mandeep Kaur, Asgar Farahnaky, Peter Joseph Torley, Andrew Mark Osborn

**Affiliations:** Discipline of Biosciences and Food Technology, School of Science, RMIT University, Melbourne 3000, Australia; s3826392@student.rmit.edu.au (S.M.H.M.); mandeep.kaur@rmit.edu.au (M.K.); asgar.farahnaky@rmit.edu.au (A.F.); mark.osborn@rmit.edu.au (A.M.O.)

**Keywords:** modified atmosphere packaging, CO_2_ emitter pad, meat spoilage, meat quality

## Abstract

Over 21 days of cold storage, the quality and microbial composition of beef steaks in response to different high-CO_2_ packaging conditions achieved by flushing gas mixtures or embedding gas emitters into the packages were studied. The results revealed that the high levels of CO_2_, achieved by either the gas flushing or the CO_2_ emitter pads, effectively controlled the number of aerobic counts. The headspace CO_2_ increased quickly in response to using the CO_2_ emitter pads, and the meat samples presented different pH levels and surface color (a* and b*) values compared to the samples packaged with the gas flushing technique. Excessive accumulation of gas in the packages that contained CO_2_ emitters resulted in package swelling and higher levels of drip loss. The longest overall quality and attractive red color of the meat samples were observed when the packages were initially flushed with the headspace gas mixture containing high levels of oxygen. Overall, using CO_2_ emitters for meat packaging can be suggested when a topfilm with proper permeability to O_2_ and CO_2_ gases is used to regulate the internal CO_2_/O_2_ and gas/product ratios.

## 1. Introduction

Fresh meat is normally a very perishable product with a limited shelf life. Containing high water content and a range of nutrients such as sugars, amino acids, nucleotides and peptides makes meat a product prone to microbial spoilage [1]. A combination of microbial and biochemical changes including discoloration [2], pH change [3] and texture degradation [4] will start in response to packaging and storage conditions, and spoiled meat is normally characterized by development of unacceptable color, odors and flavors together with slime formation [5].

In the meat ‘case-ready’ market, beef cuts are packaged in different ways including modified atmosphere packaging (MAP), vacuum packed, skin packed or being overwrapped on polystyrene trays [6]. The effect of modified atmosphere packaging on meat shelf life is largely dependent on inhibitory effects of high-CO_2_ atmospheres on growth of a varied range of spoilage and pathogenic bacteria [7]. It has been demonstrated that the presence of at least 30% CO_2_ in package headspace can effectively reduce growth and population of many key meat-associated bacteria including Enterobacteriaceae [8], *Pseudomonas* spp. [9], *Brochothrix thermosphacta* [10], *Lactobacillus* spp. [11], *Leuconostoc* spp. and lactic acid bacteria [12].

In designing an effective MAP system and to take advantage of the antibacterial effect of high-CO_2_ active packaging, several parameters should be considered including meat product respiration rate [13], packaging film permeability [14], headspace gas-to-product (G:P) ratio [15] and also solubility of CO_2_ gas within the product [16]. Following packaging, part of the headspace CO_2_ gradually dissolves within meat surface layers to form carbonic acid that subsequently dissociates into bicarbonate (HCO_3_^−^) and hydrogen (H^+^) ions [17]. Accumulation of protons can directly or indirectly influence meat quality parameters such as pH [18], color [19], drip loss [20] and water holding capacity [21] as well as cooking flavor and texture [22].

Currently, to achieve an effective modified atmosphere environment for meat products, the trays are flushed with a mixture of N_2_, O_2_ and CO_2_ gases before the topfilm is sealed [23]. Commercially, a mixture of 60–80% O_2_ and 20–30% CO_2_ is used to produce an attractive red color in the meat and to retard the growth of spoilage microorganisms [7]. The requirements for gas flushing (i.e., the need for food-grade gases, specialized mixing equipment and precise metering tools to ensure the right balance of gas mixture) make this method expensive and difficult to implement in some situations (e.g., small-scale meat processing and packaging companies). Furthermore, due to the high solubility of CO_2_ gas in meat (ca. 1 L.kg^−1^) an initial G:P ratio of 2:1 to 3:1 is required to prevent deformation and collapse of the packages following gradual absorption of the headspace gases into the meat during storage time [24]. The resulting bigger package volume leads to increased costs due to the need for more chilled storage and transportation space [25].

Recently, a number of CO_2_ emitter pads have been developed to be used in meat packages. These emitter pads commonly contain a mixture of sodium bicarbonate and citric acid (1:1 *w*:*w*), which releases CO_2_ upon receiving meat exudates [26]. The effect of CO_2_ emitter pads in prolonging the shelf life of some types of meat products such as fresh fish fillets [27,28], chicken fillets [29] and reindeer meat [30] have been previously reported. Whilst this prior research demonstrates the potential efficacy of these CO_2_ emitting pads for different packaged-meat types, there is a gap of data on the efficiency of these active pads compared to conventional gas flushing methods on physicochemical and microbiological qualities of beef meat. The purpose of this research was to compare the responses of fresh beef cuts in terms of quality parameters and spoilage bacteria throughout storage time in different high-CO_2_ active packaging treatments gained by flushing with a known gas mixture or placing different types of CO_2_ emitter pads under the meat.

## 2. Materials and Methods

### 2.1. CO_2_ Emitter Pads and Meat Samples: Selection and Suppliers

Two different commercial CO_2_ emitter pads were obtained from Vartdal Plast (VP), Vartdal, Norway and McAirlaid’s (MA), Berlingerode, Germany. The sizes for VP and MA pads were 10 cm × 15 cm and 8 cm × 13 cm, respectively. Both pad types are designed to release CO_2_ gas upon contact with water or meat exudate, causing a chemical reaction by the combination of citric acid and sodium bicarbonate. Passive pads (PP), 8 × 13 cm in size with no CO_2_ emitting chemicals, were used as negative control. The meat samples (beef semitendinosus) were purchased from a local meat wholesaler (PJ Meats, Melbourne, Australia) on the day of the experiment. The wholesaler cut the muscles into uniform ~2 cm-thick slices. The meat slices were transferred immediately to the Food Research and Innovation Centre at RMIT University.

### 2.2. CO_2_ Emitter Pads, Gas Flushing, and Meat Sample Preparation

**Figure 1:**
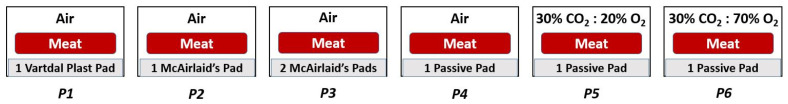
Illustration of the packaging treatments.

To create the six different active and passive packaging conditions illustrated in Figure 1, a T-200 Multivac (Keilor Park, Australia) equipped with a gas flushing system was used. For each treatment, 18 packaging trays (1L PP, Cryovac^®^) (Fawkner, Australia) were prepared and the appropriate number/type of VP, MA and PP were put at the bottom of trays (1 VP pad for P1, 1 MA pad for P2, 2 MA pads for P3 and 1 passive pad for P4, P5, P6). Immediately after placing 250 ± 2 g beef meat slices on the pads, the trays were flushed with the selected headspace gas composition and sealed with biaxially oriented polyamide/ethylene vinyl alcohol copolymer polyethylene film (oxygen transmission rate ≤ 4 cm^3^/m^2^–24 h-atm at 38 °C in dry air; LID-AEE-AP-45, Multivac, Wolfertschwenden, Germany).

Three separate replications were considered for each treatment at each analysis time. Packaged meat trays were stored at 4 °C, and analyses of samples were performed at days 3, 7, 10, 14, 17 and 21 for meat quality and microbiology counts. Untreated samples were used as purchased for day 0 analysis.

### 2.3. CO_2_ Emitter Pads and Meat Sample Analyses

Gas composition: Before opening each package, the headspace gas composition was measured using a portable O_2_/CO_2_ gas analyzer (Quantek Model Q2, Grafton, MA, USA). This was accomplished by inserting the gas analyzer needle through the top film, then drawing a gas sample from the pack’s headspace into the gas analyzer through the connecting plastic hose.

Surface color: Ten minutes after opening each tray, the surface colors of both the top side (air facing) and pad side (bottom facing and in contact with the pad) of beef slices was evaluated based on the CIE Lab system using a Chroma Meter (CR-400, Konica Minolta, Tokyo, Japan). Six readings for lightness (L^∗^), redness (a^∗^) and yellowness (b^∗^) were taken on each side of the meat slice, and the mean values were used for data analysis.

Determination of pH: Each meat sample was cut into two halves (pad side and top side) using a sharp knife. From each side, 10 g was removed and placed in a plastic beaker. After adding 10 mL distilled water, a slurry was made using an overhead stirrer (Ultra Turrax T25—IKA, Staufen, Germany) at 8000 rpm for 1 min. The Oakton pH 700 Benchtop Meter (Oakton Instruments, Vernon Hills, IL, USA) was first calibrated according to manufacturer’s instructions for two-point calibration with pH 4.01 and 7.01 buffer solutions and then used for measuring the pH of prepared meat slurries [31].

Tray swelling/collapse: To understand the effect of the interaction of meat, pad and headspace gas on the package swelling or collapse, the increase or decrease in the package height compared to day 0 was measured with a metal ruler (millimeter markings) at each sampling time point.

Drip loss: The weight of the meat slices was recorded on day 0 (D_0_) and after removal from package on the day of sampling and quality analysis (D_a_). Prior to weighing, the samples were gently blotted using tissue paper. The equation below, Equation (1), was used to calculate the percentage drip loss [32].

% Drip Loss = (D0 − Da)/D0 × 100(1)

Bacterial Counts: Total aerobic, Enterobacteriaceae, *Brochothrix thermosphacta*, Pseudomonas and lactic acid bacterial counts were determined at days 0, 3, 7, 10, 14, 17 and 21. According to the method described in ISO 6887-2:2017 [33], a section of the meat surface (5 cm^2^) was removed from each meat sample, weighed and placed in Interscience 400 mL Filter Bags (Edwards, Herblay-sur-Seine, France). According to the sample weight, 10× sterile 0.1% (*w*/*v*) peptone solution (LP0037, Oxoid, Basingstoke, UK) was added to each bag, and the meat was homogenized in a stomacher (BagMixer, Bernolsheim, France) for 2 min. A volume of 1 mL of the filtered suspension was used to generate a ten-fold dilution series [34].

Total aerobic (AC), Enterobacteriaceae (EB) and lactic acid bacteria (LAB) counts were determined using 3M™ Petrifilm Rapid Plates (St. Paul, MN, USA) (Codes 6478, 6421 and 6461, respectively). Briefly, the Petrifilm plates were placed on a flat surface, the top film lifted, 1 mL of sample suspension was dispensed onto the center of the bottom film and then the top film was gently dropped down to the sample. A spreader was used to distribute the sample evenly on the plate growth area. After allowing 1 min to form the gel, the plates were incubated in a horizontal position with the clear side up for 24 h at 30 °C (for EB) or 4 days at 25 °C (for AC and LAB). To determine the numbers of *Pseudomonas* spp. and *B. thermosphacta*, agar-based plates were used. From appropriate dilutions, 0.1 mL aliquots were spread onto *Pseudomonas* agar base (CM0559, Oxoid, UK) and onto streptomycin thallous acetate actidione agar (CM0881, Oxoid, UK); inoculated plates were incubated at 25 °C for 48 h.

The number of Colony Forming Units (CFU) for all agar-based plates and Petrifilm Rapid plates were counted using a standard colony counter, and microbiological data were expressed as Log10 CFU/cm^2^.

### 2.4. Statistical Analysis

The data of both microbial and physical parameters were subjected to one-way analysis of variance (ANOVA) to explore the significant differences among packaging treatments, storage duration and packaging–time interaction. The ANOVA was performed using MSTAT-C 8.0 for randomized complete block design (two factors), and the differences between the means (*p* < 0.05) were determined by the least significant difference (LSD test).

## 3. Results and Discussion

### 3.1. Headspace CO_2_ and O_2_ Levels

**Figure 2:**
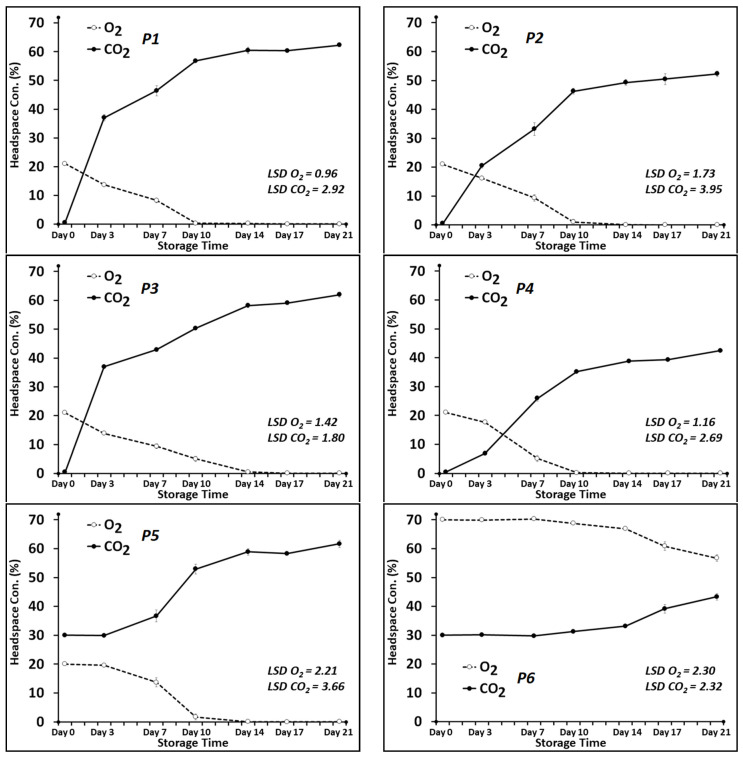
Headspace gas compositions of the packaged beef slices over 21 days of storage at 4 °C. P1, 1 VP pad/air as headspace; P2, 1 MA pad/air as headspace; P3, 2 MA pads/air as headspace; P4, 1 passive pad/air as headspace; P5, 1 passive pad/30% CO_2_: 20% O_2_: 50% N_2_ as headspace; P6, 1 passive pad/30% CO_2_: 70% O_2_ as headspace.

Throughout the storage time, the headspace concentrations of CO_2_ and O_2_ gases changed significantly (*p* ≤ 0.05) in all the treatments. The treatments which contained CO_2_ emitter pads, i.e., P1, P2 and P3, experienced the most rapid changes in the headspace gas composition (Figure 2). After 3 days, CO_2_ sharply increased to ~37% in P1 and P3 and ~20% in P2. The rising trend for headspace CO_2_ continued for all the treatments throughout the study time and rose to around 60% (for P1, P3, P5), 50% (for P2) and 40% (for P4 and P6) after 21 days of cold storage. A comparison of the two different commercial CO_2_ emitter pads, i.e., VP (P1) and MA (P2), clearly showed that the VP pads, which were 1.4 times bigger in size, had considerably higher rates of CO_2_-release capacity compared to the MA pads. For P3, when two MA pads were used, the pattern of the headspace CO_2_ changes was fairly similar to P1, using one VP pad.

Oxygen is an important gas in terms of its effect on respiratory behaviors of meat and associated microbes as well as on oxidative reactions which determine meat color. The initial concentrations of O_2_ were 70% for treatment P6 and 21% (ambient air concentration) for the other treatments. With the exception of the P6 treatment, the levels of oxygen dropped quickly for all the treatments and fell to around zero after 10 days (for P1, P2, P4, P5) or 14 days (for P3) of cold storage. Dynamic behavior of headspace gases under modified atmosphere packaging is well documented, and it is attributed to many parameters including food respiration rate, associated microbial respiration, G:P ratio and gas permeability of the packaging film [35].

The exact amount of CO_2_ generated by meat/associated microbiome cannot be monitored easily, as gaseous CO_2_ is highly soluble into the water phase of meat products [36]. In this current study, only treatment P6 maintained a balanced level of CO_2_ and O_2_ in the package over the entire storage time. For the treatments containing the CO_2_ emitter pads (P1, P3 and P2), the rapid increase of CO_2_ to over 30% and the depletion of O_2_ to near zero can primarily be explained by the production of high volumes of CO_2_ gas by the emitter pads. Previously, the activity of spoilage bacteria associated with fresh meat, such as *Brochothrix thermosphacta*, *Pseudomonas* spp., and varied range of LAB, has been reported as utilizing the available O_2_ and emitting more CO_2_ into the package headspace [37,38,39]. Additionally, ‘high barrier’ packaging materials (trays and lidding materials) could be considered as factors in limiting oxygen transmission when in-pack O_2_ quickly falls to the very low concentrations where anaerobic respiration initiates [7].

Choosing a topfilm with reasonable levels of permeability can induce a desirable equilibrium modified atmosphere (EMA) when the transmission rates of O_2_ and CO_2_ through the film equal the rates of generated/consumed gases in the tray, achieving a successful MAP using CO_2_ emitter pads.

### 3.2. Color

**Figure 3:**
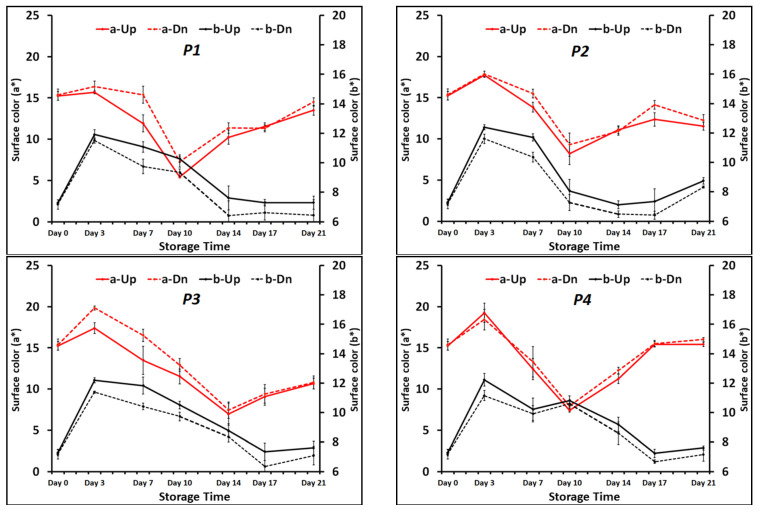

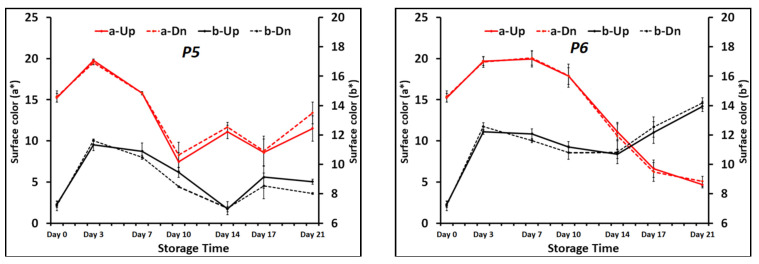
The a* values (red lines) and b* values (black lines) of top sides (solid lines) and pad sides (dashed lines) of meat samples under applied treatments. P1, 1 VP pad/air as headspace; P2, 1 MA pad/air as headspace; P3, 2 MA pads/air as headspace; P4, 1 passive pad/air as headspace; P5, 1 passive pad/30% CO_2_: 20% O_2_ as headspace; P6, 1 passive pad/30% CO_2_: 70% O_2_ as headspace.

Meat color is one of the major features that determines consumer acceptance of meat products at the point of sale [8]. The color CIE a* (red–green) and b* (yellow–blue) values for both sides (pad side and top side) of the beef steaks are presented in Figure 3. Results showed that in the treatments containing the CO_2_ emitter pads (P1, P2 and P3), the pad sides (which were in contact with the pads) and the top sides of the meat samples had significantly different values for both a* and b*, and the parts of the meat which were in contact with the CO_2_ emitter pads had lower b* values and higher a* values throughout the storage time. The largest differences were recorded for the P1 and P3 treatments at day seven, where the a* values of the pad sides (15.4 and 16.5, respectively) were more than three units higher, and the b* values of the pad sides (11.1 and 11.8, respectively) were around 1.3 units lower than the top sides’ values. For other treatments (i.e., P4, P5 and P6), no differences were found between the two sides of the meat samples. Holman et al. [40] demonstrated that a* value will provide the simplest and strongest prediction of beef color acceptability by customers. Pertaining to the b* values, however, an increase from the initial value of 7.3 happened for all the treatments, but almost all of them (except P6) followed the same pattern of changes over the 21 days of storage.

Initial L* value of the meat samples at day 0 was 36.14. For the first three days of storage, all the treatments exhibited a slight increase in the L* values (*p* < 0.05), but there was not any notable difference between the applied treatments, and the L* value fluctuated between 40 and 50 for all the samples throughout the storage period.

The a* value is an indicator of the red color of meat. The most acceptable red color and the lowest alteration of a* value was noted for treatment P6. Presence of high levels of O_2_ (60–80%) in a MAP can produce high levels of OxyMb on meat surfaces, which provide an attractive red color for short-term retail display [7]. After day 10, the red color and consequently the a* value of P6 dropped dramatically. The quick fading of the red color can be attributed to the stimulation of lipid and protein oxidation and consequent premature browning of the meat, which is detrimental to meat flavor, texture and color [41].

For the other treatments, the a* value (or red color) decreased gradually to around seven until day 10 or 14 and then experienced increases with different rates. These increases can be explained by formation of DeoxyMb pigment and development of purple-red color in the absence of enough headspace oxygen [42].

### 3.3. pH

Meat pH is believed to be a physiological parameter which reacts quickly in response to most post-slaughter conditions. The pH changes of the meat samples for both the top sides and the pad sides over 21 days of cold storage are presented in Table 1. The results revealed that the treatments which contained the CO_2_ emitter pads (P1, P2 and P3) followed a similar pattern of pH change with significantly higher values for pad sides for the majority of storage time. In those treatments, pH increased from the initial value of 5.3 to around 5.8 on day 14, and then slightly decreased for the rest of storage time. In P4, the pH did not fluctuate significantly, and the maximum pH value (5.7) was recorded on day 7. In P5, the pH increased gently until day 14 (pH 5.7) and then decreased to pH 5.4 for the pad side on day 21. An entirely different pattern of pH alteration was recorded for the treatment P6 which was almost stable for the first 10 days and then dropped sharply to around pH 5.1 by day 21.

Many studies have been conducted regarding pre- and post-slaughter meat pH and impacts of meat type, packaging condition and storage time on variations of pH. Normally, microbial contamination and spoilage of meat starts from outer layers and any changes in pH of these layers is of great importance. Carse and Locker [43] examined surface pH of sheep and beef carcasses stored at 5 °C for 5 days. Over the first three days, the pH of both meat types tended to be stable, but in the subsequent two days some locations started to show a decrease or increase in pH. Conte-Junior et al. [7] applied different levels of headspace CO_2_ (10, 20, 30, 40 and 50%) for packaging ground beef, and they did not report any difference in pH of the meat samples within 20 days of storage at 2 °C. In Australia, according to Meat Standards Australia (MSA) the optimum pH level for meat certification is 5.7 and below [44].

As presented in Table 1, for the treatments containing the CO_2_ emitter pads, the meat pH exceeded this limit when it reached pH 5.9 at day 7 (for the pad side of P1) or day 10 (for both sides of P2 and the pad side of P3). The most stable pH was found for treatment P6, where the meat samples were packaged with the passive pads under 30% CO_2_: 70% O_2_. The initial increases in the levels of pH for the P1, P2 and P3 treatments could be attributed to the accumulation of basic compounds resulting from the activity of specific bacterial groups such as *Brochothrix thermosphacta* and *Pseudomonas* spp., and the later reductions of pH can be explained by dissolution of the headspace CO_2_ in the meat, formation of carbonic acid and gradual release and accumulation of H^+^ in the meats’ water [45]. Our results were in accordance with Muhlisin et al. [46], who examined the quality of beef meat in modified atmosphere packaging under 20% CO_2_ in combination with 0, 30 or 70% O_2_ and reported a gradual increase of meat pH over 12 days of cold storage. According to Borch et al. [47], fluctuation of meat pH during storage is a complex interaction between muscular glucose resources, glycolytic enzymes and activity of different bacterial groups.

### 3.4. Microbiological Examination

Except Enterobacteriaceae which was not detected in any of the treatments throughout the storage time, the data of the viable counts for the other microbial groups including total aerobic count, lactic acid bacteria, *Brochothrix thermosphacta* and *Pseudomonas* spp. from the meat samples under the different packaging conditions are presented in Table 2. The low number of counts from each microbial group at day zero indicates that good hygiene was practiced during preparation and packaging. Among the applied treatments, P1 and P3, which contained 1 VP and 2 MA CO_2_ emitter pads, respectively, plus P5, which contained 30% flushed CO_2_ initially, controlled the number of aerobic bacteria effectively. Treatment P2, which contained one MA pad only, recorded a higher number of bacterial growth for almost all the bacterial groups studied. For treatment P4 which had no CO_2_ emitter pad and an initial headspace of ambient air, a sharp increase in the number of aerobic counts (from 2.3 to 4.3 Log CFU/cm^2^) was recorded within the first 3 days of storage, and this growth continued to the end of the experiment and reached 6.3 Log CFU/cm^2^ after 21 days. This data revealed that high levels of CO_2_ in packages from the initial hours of storage can significantly lower the activity of aerobic bacteria. Gill and Gill [48] stated that presence of 20% CO_2_ in the headspace of meat packages can reduce growth of psychrotrophic microorganisms by 50%. Wang et al. [49] indicated that initial presence of 20, 60 and/or 100% CO_2_ in packages effectively inhibited growth of viable bacteria and delayed bacterial spoilage of smoked chicken legs.

Lactic acid bacteria (LAB) are believed to contribute to meat spoilage when there is an increased level of CO_2_ in packages [50]. In this experiment, the numbers of LAB grew quickly in the P6 treatment (30% CO_2_: 70% O_2_ initially) compared to the other treatments and increased from the initial level of 1.8 to 5.0 Log cfu/cm^2^ at day 5 and finally to 7.1 Log cfu/cm^2^ after 21 days of cold storage. Today, so-called high-O_2_ MAP (70–80% O_2_: 20–30% CO_2_) is mainly used for short-term retail packaging of fresh meat. According to the results in Table 2, some members of lactic acid bacteria can perform aerobic respiration making them facultative anaerobes with their ability to grow under high levels of CO_2_. Similar growth in numbers of LAB on minced buffalo meat under high-O_2_ MAP (80% O_2_: 20% CO_2_) was reported by Jaberi et al. [51].

For the first two weeks of storage, the P3 and P5 treatments kept their LAB contamination (5.3 and 5.5 Log cfu/cm^2^, respectively) at an acceptable threshold, and using CO_2_ emitter pads in P1 and P2 did not show any meaningful effect on LAB counts. Presence of a flora of psychrotrophic lactic acid bacteria on fresh chilled meats usually ensures extended shelf life [52]. Overall, in the treatments containing the CO_2_ emitter pads, the presence of LAB communities did not result in quality defects up to day 14 of the storage, and spoilage symptoms including slime formation were not detected.

*Brochothrix thermosphacta* is one of the most frequent spoilage bacteria of fresh meats, due to its tolerance to low-pH conditions and growth at refrigeration temperatures [53]. Among the applied treatments, P6 presented the lowest *B. thermosphacta* levels with no colonies for the first seven storage days, and a slow increase to 2.0 Log cfu/cm^2^ after 21 days of cold storage. For the CO_2_ emitter pad-containing treatments (P1, P2 and P3), a similar pattern of changes and moderate numbers of *B. thermosphacta* colonies were recorded. Treatment P4, as the control with no CO_2_ releasing pad and no initial flushing CO_2_, presented the quickest increase in *B. thermosphacta* colonies (3.4 Log cfu/cm^2^ on day 7). Thus, the activity of this fermentative bacteria can be considered as a reason for the sharp increase in the headspace CO_2_ in this treatment (Table 2). Presence of *B. thermosphacta* on meat samples even when there is no oxygen in packages can be explained by the capability of this bacterium to grow both aerobically and anaerobically which makes it a substantial meat-spoilage flora [11].

Several *Pseudomonas* spp. are recognized as causative agents of meat spoilage under aerobic conditions. These proteolytic bacteria can activate in a wide range of temperatures (−1 to 25 °C) and commonly break down proteins and produce a variety of odors and flavors under extended refrigerated storage [10]. The initial level of *Pseudomonas* contamination on day 0 was around 2 Log cfu/cm^2^. In the P1, P2 and P3 treatments (the trays containing the CO_2_ emitter pads), *Pseudomonas* increased slightly throughout the storage time and fluctuated between 2–3 Log cfu/cm^2^. Treatment P5, which started with 30% CO_2_: 20% O_2_, followed the same pattern. The most changes and the highest number of *Pseudomonas* counts were recorded for treatment P4 followed by P6. Treatment P4 was the only treatment which started with zero additional CO_2_ in the trays, and it seems that in the absence of additional CO_2_ and its inhibitory effect and before developing a number of LAB as competitive bacteria, *Pseudomonas* spp. had enough time to grow and deteriorate the P4 meat samples.

### 3.5. Tray Swelling/Collapse

**Figure 4:**
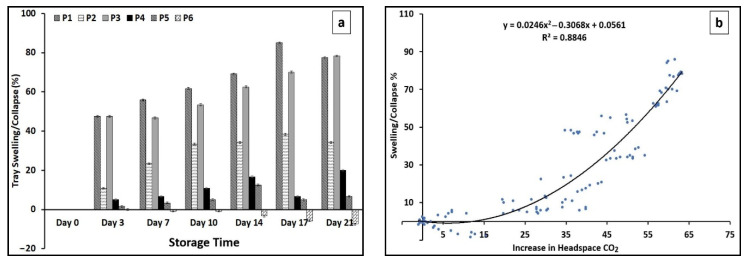
The rates of tray swelling/collapse ± SE (**a**) and the correlations between swelling/collapse percent and increase in the headspace CO_2_ (**b**). P1, 1 VP pad/air as headspace; P2, 1 MA pad/air as headspace; P3, 2 MA pads/air as headspace; P4, 1 passive pad/air as headspace; P5, 1 passive pad/30% CO_2_: 20% O_2_ as headspace; P6, 1 passive pad/30% CO_2_: 70% O_2_ as headspace.

Blown packaging over storage times has been previously reported for vacuum-packaged beef and lamb meat [54]. Swollen or puffy-looking packages are considered a sign of CO_2_ gas release as a result of excessive growth of some types of bacteria such as psychrophilic *Clostridia* spp. [55]. As presented in Figure 4a, an increasing rate of package swelling throughout the storage period was recorded for almost all the treatments (except P6). The highest values of package swelling were found for treatments P1 (85%) and P3 (78%), followed by P2 (38%). This means the treatments containing the CO_2_ emitter pads experienced excessive accumulation of CO_2_ inside the packages. We used an EVOH (ethyl vinyl alcohol) with polyethylene (PE)-based film as the sealing topfilm. This film is widely used in creating MAP because of its excellent gas barrier, having high thermal resistance and high puncture resistance as well as good optical characteristics. The impermeability of the topfilm contributed to the accumulation of gases by trapping the released gases inside the package. Figure 4b, shows the linear correlation between the percentage of package swelling and the increase in the concentration of CO_2_ in packages which clearly confirms the role of the CO_2_ emitter pads in the swelling of the packages. Up to around ± 10%, changes in the package height normally didn’t affect the package shape significantly. Between the applied treatments, P5 with around 12% increase (swelling) and P6 with 7% decrease (collapse) in the tray height maintained the acceptable package shape after 21 days of storage. Collapse in meat packages normally happens when part of the headspace gases, mainly CO_2_, is absorbed by the meat [56]. Since interactions between product, film permeability and package size determine gas compositions inside packages, more research is required to predict the dynamics of gas exchange of packages throughout storage periods [57].

### 3.6. Drip Loss

**Figure 5:**
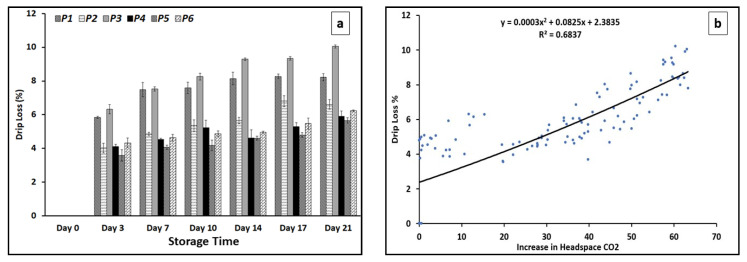
Drip loss formation ± SE (**a**) and the correlation between drip loss % and increase in headspace CO_2_ (**b**). P1, 1 VP pad/air as headspace; P2, 1 MA pad/air as headspace; P3, 2 MA pads/air as headspace; P4, 1 passive pad/air as headspace; P5, 1 passive pad/30% CO_2_: 20% O_2_ as headspace; P6, 1 passive pad/30% CO_2_: 70% O_2_ as headspace.

In general, drip loss is a natural event, referring to loss of intramuscular fluids from meat due to gravitational forces. Water remaining in the meat is one of the most important traits of meat quality [58]. As expressed in Figure 5a, the highest rates of drip loss were recorded in the CO_2_ emitter pad-containing treatments (P1 and P3 followed by P2). The majority of the drip loss occurred during the first three days of storage (~6% for P1 and P3 and ~4% for P2, P4, P5 and P6) with a gradual further loss throughout the remainder of the storage time.

A negative effect of storage time on drip loss has been demonstrated previously [59]. The correlation between the increase in the headspace CO_2_ and the drip loss (Figure 5b) demonstrated the impact of the high levels of CO_2_ accumulation in the packages on the drip loss. In the CO_2_-releasing pad treatments, the accumulation of CO_2_ gas inside the packages put pressure on the package walls and the meat samples inside and resulted in the larger values of drip loss from the samples. The drip loss values in the beef meat for the P4, P5 and P6 treatments in the current study were higher than those found by Payne et al. [60] for different non-vacuum chilled beef packages but were in the range reported by Hur et al. [31] for beef meat under modified atmosphere packaging.

## 4. Conclusions

In conclusion, these results showed that using CO_2_ emitter pads could effectively control the number of aerobic counts. The highest number of aerobic counts, *Pseudomonas* and *Brochothrix thermosphacta* of the meat samples were detected in the P4 (control) where there was neither an emitter pad nor initial CO_2_ flushed to the trays. The sharp decreases in the headspace O_2_ and the excessive package swelling due to the accumulation of CO_2_ revealed that among the several controlling parameters in designing a successful MAP, the permeability of the packaging film to O_2_ and CO_2_ gases is very important in keeping a balanced gas–product (G:P) ratio, O_2_: CO_2_ ratio and package shape throughout storage periods. Keeping a minimum level of oxygen alongside a balanced level of high CO_2_ is critical in preserving attractive meat color, controlling microbial spoilage and increasing the shelf life of fresh meat. CO_2_ emitter pads could successfully replace the gas flushing system if they are used with an appropriate topfilm. More importantly, there is more work required to adjust the CO_2_-releasing capacity of gas emitters to meat type, meat and package size and the product’s expected shelf life.

## Figures and Tables

**Table 1:** The pH values of both beef sample sides during 21 days of cold storage under different packaging conditions.

Code	Packaging	Meat Side	Storage Time						
	(Headspace/Pad)		Day 0	Day 3	Day 7	Day 10	Day 14	Day 17	Day 21
P1	Air/1 VP	Top	5.3 ± 0.0	5.6 ± 0.0	5.7 ± 0.0	5.8 ± 0.1	5.8 ± 0.0	5.8 ± 0.0	5.7 ± 0.0
		Down	5.3 ± 0.0	5.6 ± 0.1	5.9 ± 0.0	5.9 ± 0.0	6.0 ± 0.1	5.9 ± 0.0	5.7 ± 0.0
P2	Air/1 MA	Top	5.3 ± 0.0	5.6 ± 0.0	5.7 ± 0.0	5.8 ± 0.0	5.8 ± 0.0	5.7 ± 0.0	5.7 ± 0.0
		Down	5.3 ± 0.0	5.6 ± 0.0	5.7 ± 0.0	5.9 ± 0.0	5.9 ± 0.0	5.8 ± 0.1	5.6 ± 0.0
P3	Air/2 MA	Top	5.3 ± 0.0	5.6 ± 0.0	5.7 ± 0.1	5.7 ± 0.1	5.8 ± 0.0	5.9 ± 0.0	5.7 ± 0.0
		Down	5.3 ± 0.0	5.7 ± 0.0	5.7 ± 0.0	5.9 ± 0.1	5.9 ± 0.0	5.9 ± 0.0	5.7 ± 0.0
P4	Air/1 PP	Top	5.3 ± 0.0	5.6 ± 0.1	5.7 ± 0.1	5.6 ± 0.0	5.6 ± 0.1	5.6 ± 0.1	5.5 ± 0.0
		Down	5.3 ± 0.0	5.6 ± 0.0	5.7 ± 0.1	5.6 ± 0.0	5.6 ± 0.0	5.5 ± 0.1	5.5 ± 0.0
P5	30% CO_2_: 20% O_2_/1 PP	Top	5.3 ± 0.0	5.6 ± 0.0	5.6 ± 0.0	5.7 ± 0.0	5.7 ± 0.0	5.6 ± 0.0	5.5 ± 0.0
		Down	5.3 ± 0.0	5.6 ± 0.0	5.6 ± 0.0	5.7 ± 0.0	5.7 ± 0.1	5.6 ± 0.0	5.4 ± 0.0
P6	30% CO_2_: 70% O_2_/1 PP	Top	5.3 ± 0.0	5.5 ± 0.0	5.6 ± 0.0	5.6 ± 0.0	5.3 ± 0.0	5.3 ± 0.0	5.1 ± 0.0
		Down	5.3 ± 0.0	5.6 ± 0.0	5.6 ± 0.0	5.6 ± 0.1	5.3 ± 0.0	5.2 ± 0.0	5.1 ± 0.0

**Table 2:** Viable counts of different bacterial groups on meat surfaces (Log CFU/cm2 ± SE) during 21 days of cold storage under different packaging conditions.

	Code	Packaging (P)	Storage Time (T)							LSD
		(Headspace/Pad)	Day 0	Day 3	Day 7	Day 10	Day 14	Day 17	Day 21	(S × T)
Aerobic Counts	P1	Air/1 VP	2.3 ± 0.2	2.7 ± 0.1	2.8 ± 0.2	3.9 ± 0.2	5.1 ± 0.2	5.4 ± 0.0	5.5 ± 0.1	0.63
	P2	Air/1 MA	2.3 ± 0.2	3.0 ± 0.2	3.1 ± 0.5	4.2 ± 0.1	5.2 ± 0.1	5.8 ± 0.1	5.2 ± 0.5	
	P3	Air/2 MA	2.3 ± 0.2	2.4 ± 0.3	2.8 ± 0.3	3.6 ± 0.5	5.1 ± 0.1	5.6 ± 0.1	5.7 ± 0.4	
	P4	Air/1 PP	2.3 ± 0.2	4.3 ± 0.4	4.3 ± 0.2	4.6 ± 0.0	5.8 ± 0.1	5.9 ± 0.1	6.3 ± 0.1	
	P5	30% CO_2_: 20% O_2_/1 PP	2.3 ± 0.2	2.5 ± 0.1	2.6 ± 0.1	3.6 ± 0.5	4.7 ± 0.1	5.5 ± 0.2	5.7 ± 0.3	
	P6	30% CO_2_: 70% O_2_/1 PP	2.3 ± 0.2	3.5 ± 0.1	3.5 ± 0.2	4.3 ± 0.1	4.8 ± 0.4	6.4 ± 0.1	6.4 ± 0.1	
Lactic acid bacteria	P1	Air/1 VP	1.8 ± 0.1	2.1 ± 0.2	3.8 ± 0.3	5.2 ± 0.2	5.9 ± 0.1	6.4 ± 0.0	6.8 ± 0.1	0.62
	P2	Air/1 MA	1.8 ± 0.1	2.1 ± 0.1	3.7 ± 0.2	5.4 ± 0.3	5.5 ± 0.2	5.9 ± 0.2	6.6 ± 0.1	
	P3	Air/2 MA	1.8 ± 0.1	1.9 ± 0.3	3.5 ± 0.5	4.4 ± 0.5	5.3 ± 0.1	6.0 ± 0.1	6.5 ± 0.1	
	P4	Air/1 PP	1.8 ± 0.1	2.8 ± 0.4	4.3 ± 0.2	4.9 ± 0.2	5.5 ± 0.1	5.9 ± 0.1	6.3 ± 0.2	
	P5	30% CO_2_: 20% O_2_/1 PP	1.8 ± 0.1	2.4 ± 0.4	3.5 ± 0.5	4.9 ± 0.2	5.5 ± 0.2	5.7 ± 0.2	6.1 ± 0.2	
	P6	30% CO_2_: 70% O_2_/1 PP	1.7 ± 0.1	3.2 ± 0.0	5.0 ± 0.2	5.8 ± 0.1	6.1 ± 0.1	6.7 ± 0.1	7.1 ± 0.2	
*Brochothrix* *thermosphacta*	P1	Air/1 VP	0.0 ± 0.0	0.6 ± 0.1	1.7 ± 0.0	2.4 ± 0.2	2.5 ± 0.2	2.6 ± 0.1	2.3 ± 0.1	0.91
	P2	Air/1 MA	0.0 ± 0.0	1.2 ± 0.6	2.3 ± 0.1	2.8 ± 0.0	2.7 ± 0.3	2.4 ± 0.1	2.0 ± 0.3	
	P3	Air/2 MA	0.0 ± 0.0	1.1 ± 0.5	1.6 ± 0.8	2.0 ± 0.2	2.4 ± 0.1	2.4 ± 0.1	2.4 ± 0.2	
	P4	Air/1 PP	0.0 ± 0.0	2.2 ± 0.5	3.4 ± 0.2	3.1 ± 0.1	3.0 ± 0.1	2.5 ± 0.2	2.1 ± 0.3	
	P5	30% CO_2_: 20% O_2_/1 PP	0.0 ± 0.0	0.0 ± 0.0	0.0 ± 0.0	1.4 ± 0.7	2.1 ± 0.4	2.1 ± 0.3	2.0 ± 0.1	
	P6	30% CO_2_: 70% O_2_/1 PP	0.0 ± 0.0	0.0 ± 0.0	0.0 ± 0.0	1.6 ± 0.8	1.9 ± 0.1	1.8 ± 0.0	1.9 ± 0.1	
*Pseudomonas* spp.	P1	Air/1 VP	1.7 ± 0.1	2.6 ± 0.0	2.4 ± 0.4	2.0 ± 0.1	2.2 ± 0.1	2.1 ± 0.1	2.1 ± 0.2	0.79
	P2	Air/1 MA	1.7 ± 0.1	1.9 ± 0.9	2.3 ± 0.2	2.7 ± 0.2	2.2 ± 0.2	2.4 ± 0.3	1.7 ± 0.1	
	P3	Air/2 MA	1.7 ± 0.1	1.6 ± 0.8	2.1 ± 0.3	2.2 ± 0.2	2.6 ± 0.2	2.1 ± 0.0	2.1 ± 0.3	
	P4	Air/1 PP	1.7 ± 0.1	3.4 ± 0.2	4.0 ± 0.1	3.9 ± 0.0	3.9 ± 0.1	3.7 ± 0.1	3.6 ± 0.1	
	P5	30% CO_2_: 20% O_2_/1 PP	1.7 ± 0.1	1.8 ± 0.0	1.9 ± 0.1	2.2 ± 0.3	2.4 ± 0.2	2.8 ± 0.1	2.4 ± 0.2	
	P6	30% CO_2_: 70% O_2_/1 PP	1.7 ± 0.1	2.8 ± 0.0	3.2 ± 0.1	3.8 ± 0.3	3.7 ± 0.1	3.4 ± 0.1	3.3 ± 0.0	

## Data Availability

The original contributions presented in the study are included in the article, further inquiries can be directed to the corresponding author.
